# The effects of carbohydrate-restricted diets on 24-h mean blood glucose levels measured by continuous glucose monitoring in type 2 diabetes: a hypothesis-generating meta-analysis

**DOI:** 10.3389/fnut.2025.1670022

**Published:** 2025-10-07

**Authors:** Chou Wang, Aizhen Pei

**Affiliations:** ^1^School of Sport, Health and Exercise, Loughborough University, Loughborough, United Kingdom; ^2^School of Design and Creative Arts, Loughborough University, Loughborough, United Kingdom

**Keywords:** carbohydrate-restricted diets, type 2 diabetes, meta-analysis, low carbohydrate diets, continuous glucose monitor

## Abstract

**Purpose:**

To conduct a hypothesis-generating meta-analysis exploring trends in 24-h mean blood glucose via CGM in T2DM patients on carbohydrate-restricted diets (CRDs), to inform future trial design and intervention duration.

**Methods:**

This study applied predefined inclusion and exclusion criteria and systematically searched five major databases—PubMed, Web of Science, Embase, Cochrane Library, and EBSCOhost—from their inception to May 16, 2025. The methodological quality of the selected studies was assessed in accordance with the Cochrane Handbook (version 5.1). Statistical analyses, including effect size estimation and sensitivity testing, were conducted using STATA version 18. Bias evaluation was performed using Review Manager version 5.4. Exploratory trend analyses were carried out using Microsoft Excel 2019.

**Results:**

A total of 1,322 articles were retrieved, and after four rounds of screening, seven studies involving 301 participants (mean age 58.1 ± 8.64 years) were included in the meta-analysis. Results showed that CRDs significantly improved 24-h mean blood glucose in patients with T2DM (d = −0.51, 95% CI: −0.88 to −0.14, *p* < 0.05), with exploratory trend analysis suggesting a positive correlation between intervention duration and the magnitude of 24-h mean blood glucose reduction.

**Conclusion:**

CRDs may improve 24-h MBG in patients with T2DM, with exploratory trend analysis suggesting greater benefits with longer intervention durations. However, due to the limited number and relatively short duration of included studies, further high-quality randomized controlled trials with longer durations (≥1 year) are warranted to evaluate the differential effects of short-term and long-term CRDs on glycemic outcomes in patients with T2DM.

**Systematic review registration:**

CRD420251069702, https://www.crd.york.ac.uk/PROSPERO/.

## 1 Introduction

Type 2 diabetes mellitus (T2DM) is one of the most prevalent chronic illnesses worldwide, with 536.6 million cases (10.5% of the population) reported in 2021. This number is projected to reach 783.2 million (12.2% of the population) by 2045, and health expenditures are expected to exceed 1,054 billion USD (1). Maintaining healthy blood glucose fluctuations is the key to delaying T2DM and its complications, such as kidney disease, cardiovascular disease, and retinopathy (2). The 24-h mean blood glucose (MBG) is defined as the average blood glucose concentration across a 24-h period, as measured by continuous glucose monitor (CGM) devices (3). These devices can monitor blood glucose fluctuations every 5 min. Long-term use can provide a large amount of 24-h data, which greatly facilitates research on T2DM (4). Compared to other common indicators in T2DM, such as glycated haemoglobin (HbA1c), triglycerides, and fasting plasma glucose, CGM devices provide greater advantages in terms of data collection and portability.

Exercise, pharmacological, and dietary interventions are common methods for the treatment of T2DM (5). T2DM is a metabolic disease characterized by reduced insulin sensitivity (IS) in insulin-responsive cells (6). Pharmacological treatments necessitate consistent medication use, which may result in drug dependence and adverse effects (7). Exercise interventions are flexible and convenient; however, some may pose a risk of injury to beginners and older adults (8). Dietary interventions are one of the widely recommended safe and highly efficient interventions for improving metabolic health (9, 10). Positive dietary patterns can effectively improve metabolic syndrome, and influence both disease prevention and progression (11). For example, the ketogenic diet can enhance the efficacy of phosphatidylinositol 3 kinase inhibitors and overcomes drug resistance in various cancer models by lowering blood glucose and insulin levels, thereby suppressing mTORC1 signaling (12).

Current studies present conflicting opinions regarding the effect of low carbohydrate diets on 24-h average blood glucose. Skytte et al. (13) found that compared to conventional diabetes diet, six weeks low carbohydrate diets improved postprandial glucose area under curve by 60%, 24 h glucose by 13%, postprandial insulin secretion rates by 24%, insulinogenic index by 31%, b-cell sensitivity to glucose by 45%, improving blood glucose metabolism and *β*-cell function in patient with T2DM. On the contrary, Al-Ozairi et al. (14) found that 6 days 10 and 30% low carbohydrate diets cannot decrease 24-h mean blood glucose in patients with T2DM. These findings suggest that variations in carbohydrate intake levels, duration of intervention, and participants’ body mass indexes (BMIs) may account for the differences in 24-h average blood glucose results observed in carbohydrate-restricted diets (CRDs) studies involving patients with T2DM.

Building on this background, numerous studies have investigated the effects of various dietary interventions on glucose control in patients with T2DM. For example, a meta-analysis of 10 randomized controlled trials (RCTs) involving 1,376 patients with T2DM reported a greater HbA1c reduction of 3.7 mmol/L with low-carbohydrate diets compared to high-carbohydrate diets (15). Similarly, another meta-analysis of 25 RCTs including 2,412 patients with either type 1 or type 2 diabetes found that carbohydrate restriction to less than 26% of total energy intake led to significantly greater HbA1c reductions at both 3 and 6 months, compared to moderate (26–45%) and high-carbohydrate diets (16).

Given that continuous glucose metrics such as MBG may correlate closely with HbA1c in T2DM patients, the clinical utility of MBG as an independent outcome remains debated. Therefore, this study was designed as a hypothesis-generating meta-analysis to explore preliminary patterns and trends in 24-h MBG change in response to CRDs. The findings may provide preliminary insights to inform the design of future hypothesis-testing trials and contribute to the evolving understanding of dietary management in T2DM.

## 2 Method

This meta-analysis was conducted as a hypothesis-generating exploratory analysis, aiming to investigate potential trends in 24-h MBG levels measured by CGM among patients with T2DM undergoing CRDs. The present meta-analysis was registered in the International Prospective Register of Systematic Reviews (PROSPERO) with registration number: CRD420251069702, and the full protocol is available at: https://www.crd.york.ac.uk/prospero/display_record.php?ID=CRD420251069702. This review was conducted in accordance with the Preferred Reporting Items for Systematic Reviews and Meta-Analyses (PRISMA) 2020 guidelines (17).

### 2.1 Search strategy

The search strategy was categorized into three groups, focusing, respectively, on intervention method, research subject, and outcome indicator. Each group included both search terms and free terms which were combined by OR. The three groups were combined using AND. Detailed search terms are presented in Table 1. After the literature search, the results were imported into Endnote 21 literature management software. Two researchers (CW and AP) independently screened the articles in a double blend manner, removing duplicate records by reviewing the titles and abstracts. Subsequently, researchers extracted data after retrieving the full texts. Any disagreements were resolved through discussion and consensus between the two researchers.

**Table 1:** Search terms and search formulas.

Search term classification	Search term
Intervention method	Diet [Mesh] or diets or Diet, Carbohydrate-Restricted [Mesh] or Diet, Carbohydrate Restricted or Carbohydrate-Restricted Diet or Carbohydrate Restricted Diet or Carbohydrate-Restricted Diets or Diets, Carbohydrate-Restricted or Low-Carbohydrate Diet or Diet, Low-Carbohydrate or Diets, Low-Carbohydrate or Low Carbohydrate Diet or Low-Carbohydrate Diets or Diet, Low Carbohydrate or Carbohydrate Diet, Low or Carbohydrate Diets, Low or Diets, Low Carbohydrate or Low Carbohydrate Diets
Research subject	Diabetes Mellitus, Type 2 [Mesh] or Diabetes Mellitus, Stable or Stable Diabetes Mellitus or Diabetes Mellitus, Noninsulin Dependent or Diabetes Mellitus, Adult-Onset or Adult-Onset Diabetes Mellitus or Diabetes Mellitus, Adult Onset or Diabetes Mellitus, Ketosis-Resistant or Diabetes Mellitus, Ketosis Resistant or Ketosis-Resistant Diabetes Mellitus or Diabetes Mellitus, Non Insulin Dependent or Diabetes Mellitus, Non-Insulin-Dependent or Non-Insulin-Dependent Diabetes Mellitus or Diabetes Mellitus, Type II or NIDDM or Diabetes Mellitus, Maturity-Onset or Diabetes Mellitus, Maturity Onset or Maturity-Onset Diabetes Mellitus or Maturity Onset Diabetes Mellitus or MODY or Diabetes Mellitus, Slow-Onset or Diabetes Mellitus, Slow Onset or Slow-Onset Diabetes Mellitus or Type 2 Diabetes Mellitus or Noninsulin-Dependent Diabetes Mellitus or Noninsulin Dependent Diabetes Mellitus or Maturity-Onset Diabetes or Diabetes, Maturity-Onset or Maturity Onset Diabetes or Type 2 Diabetes or Diabetes, Type 2 or Diabetes Mellitus, Noninsulin-Dependent
Outcome indicator	Continuous Glucose Monitoring [Mesh] or Glucose Monitoring, Continuous or Monitoring, Continuous Glucose or Monitorings, Continuous Glucose or Continuous Glucose Monitoring Device or CGM Device or CGM Devices or Device, CGM or Devices, CGM
Research method	RCT^a^

a RCT: randomized controlled trial.

An updated literature search was conducted up to August 2025; no additional studies meeting the predefined inclusion criteria were identified.

### 2.2 Inclusion and exclusion criteria

#### 2.2.1 Study participants

Eligible participants were adults aged 18 years or older diagnosed with T2DM. Studies were included if they implemented a structured carbohydrate-restricted dietary intervention, with low-carbohydrate diets (LCDs) defined as ≤45% of total energy from carbohydrates and very-low-carbohydrate diets defined as <26% of total energy from carbohydrates, in accordance with the American Diabetes Association consensus statement (18, 19).

#### 2.2.2 Study intervention

Studies were included only if they explicitly specified the duration of the diet and reported its effects. Only trials implementing a clearly structured carbohydrate-restricted dietary program with well-defined macronutrient composition were eligible. Trials in which meals were fully provided by the study or research-affiliated providers, thereby ensuring adherence to the prescribed dietary intervention, were prioritized. Articles that merely encouraged participants to reduce carbohydrate intake without direct supervision or provision of meals were excluded. In one study (20), participants received only key foods representing 30% of total energy consistent with the prescribed diet, with the remaining foods obtained and prepared by participants under dietitian guidance. Despite partial meal provision, adherence to macronutrient targets was maintained through the provision of key foods, individualized dietitian support, and monitoring of intake; therefore, this study was considered eligible for inclusion.

#### 2.2.3 Research comparison

A control condition without carbohydrate-restriction was required for comparison with the carbohydrate-restricted conditions. Eligible study designs included randomized comparisons, such as before-and-after studies, as well as trials using parallel or cross-over designs However, studies comparing the combined use of CRDs and exercise interventions with a control condition that did not receive same exercise interventions were deemed ineligible. For the comparator diet, studies with a defined conventional diabetes diet, typically comprising 46–60% carbohydrate, were primarily included. One crossover study (14) used a habitual diet as the comparator, for which no macronutrient composition was specified. Given the low heterogeneity (I^2^ = 0%) and robust study design, this study was included in the pooled analysis with appropriate annotation, publication bias, and sensitivity consideration.

#### 2.2.4 Measurement results

The study required data collected using CGM devices over a 24-h period under both restricting carbohydrate diets and control conditions. The outcome measure of interest was the 24-h MBG level, as measured by CGM, which was used to explore potential glycemic trends associated with carbohydrate-restricted dietary interventions.

### 2.3 Data extraction

Two researchers independently extracted relevant data from the articles:

1. Basic article data, including the first author and the year of publication.
2. Participant data, including subject population, sample size, age.
3. Details of carbohydrate-restricted diet interventions, including diet duration and levels of carbohydrate restriction.
4. Outcome indicator includes 24-h MBG measured by CGM.

### 2.4 Quality evaluation

The risk of bias for each included study was systematically appraised using Review Manager (RevMan) version 5.4, based on the methodological standards outlined in the Cochrane Handbook for Systematic Reviews of Interventions (version 5.1). The evaluation encompassed key domains: random allocation methods, allocation concealment, blinding, data outcome integrity, selective reporting, and other potential sources of bias. Each study was assigned a risk level—low, high, or unclear—according to its adherence to these criteria. In cases where information was insufficient to permit a clear judgment, the risk was deemed unclear, with explicit justification provided. Two independent reviewers conducted the assessments (CW and AP).

It should be noted that none of the included studies implemented blinding of participants, personnel, or outcome assessors. The absence of blinding may have introduced performance and detection bias, particularly in subjective outcome measures, potentially leading to an over-estimation of the intervention effect. Therefore, the pooled results should be interpreted with caution. In addition, the overall methodological quality of this systematic review was evaluated using the AMSTAR-2 tool, and the detailed results are provided in Supplementary Table 2.

### 2.5 Data analysis

Effect size synthesis was performed using STATA 18 software, and publication bias was assessed with Review Manager 5.4. All outcomes were continuous variables and reported as means ± standard deviations. Inter-group heterogeneity was evaluated using the I^2^ statistic. For outcomes with low heterogeneity (I^2^ < 50%), a fixed-effects model was applied; for substantial heterogeneity (I^2^ ≥ 50%), a random-effects model with Hartung-Knapp adjustment was used. Publication bias was assessed through funnel plot symmetry. Sensitivity analyses were conducted to verify the robustness of the results. Given the exploratory nature of this meta-analysis, the findings aim to identify potential glycemic trends rather than establish definitive clinical effects. Also, these exploratory analyses may be prone to overfitting because of the limited number of studies and variables, and results should be interpreted with caution.

Due to the limited number of included studies, formal subgroup analyses or meta-regression were not feasible. Exploratory trend analyses were conducted to investigate potential patterns in study characteristics, specifically focusing on intervention duration and the mean difference in 24-h MBG. Figures were generated using Microsoft Excel 2019, and R^2^ and *p*-values were calculated using SPSS and annotated on the graphs.

## 3 Results

### 3.1 Search results

**Figure 1:**
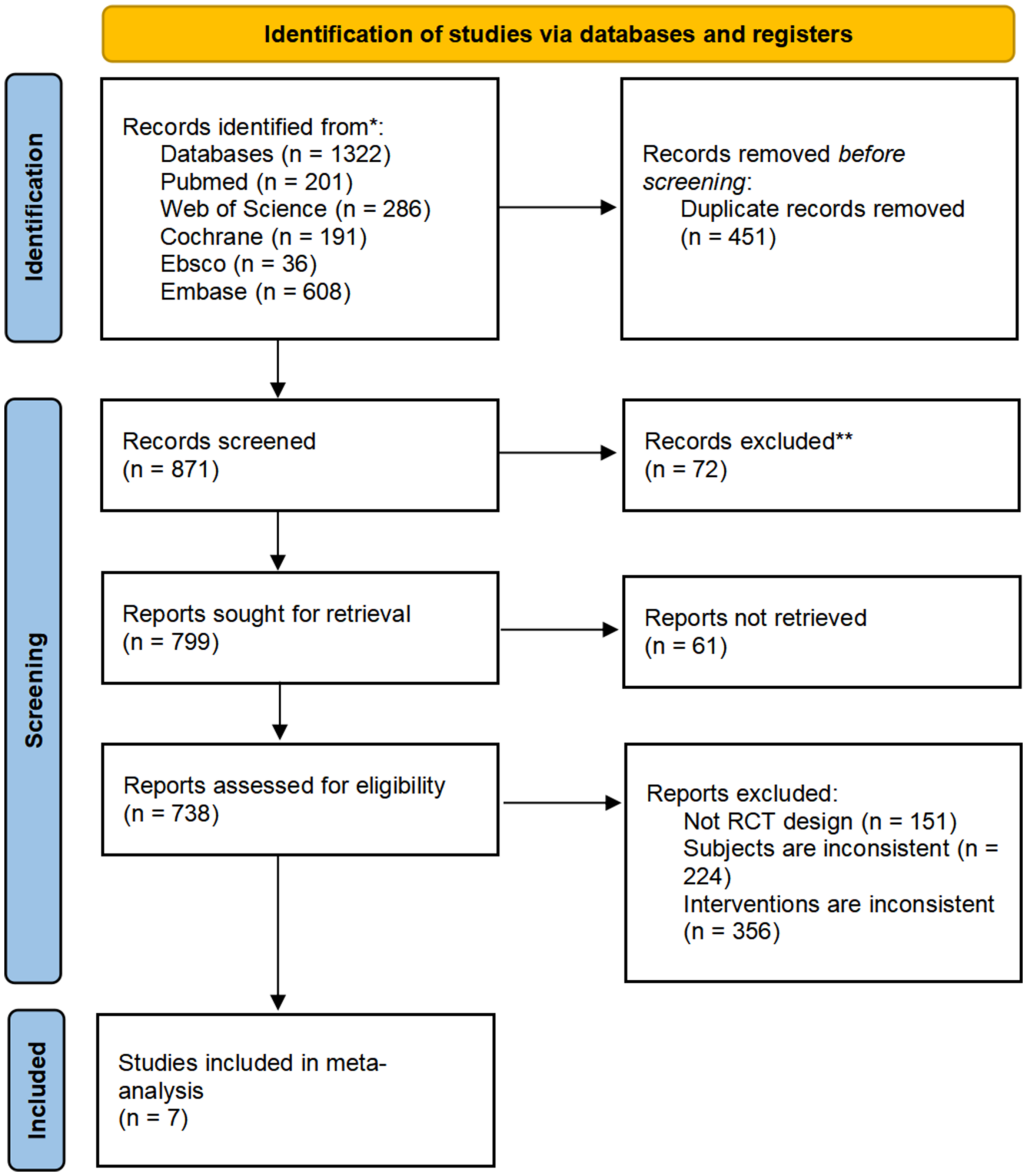
Flow chart for literature screening.

A total of 1,322 articles were retrieved: 201 from the PubMed database, 286 from the Web of Science database, 191 from the Cochrane database, 36 from the Ebsco database, and 608 from the Embase database. After screening titles, abstracts, and full texts, 1,315 articles were excluded. Ultimately, 7 studies met the inclusion criteria and were considered suitable for exploratory meta-analysis. These studies were used to investigate potential trends in the effects of CRDs on CGM-derived 24-h MBG levels in individuals with T2DM. The selection process was shown in the Figure 1.

### 3.2 Inclusion of the basic characteristics of the article

Study subjects included adult patients diagnosed with T2DM, aged 18 years or older (range: 54–64 years). The duration of the interventions ranged from 1 day to 24 weeks, with meal frequency varying from once to three times per day. The degree of carbohydrate restriction across the included studies ranged from 10 to 30% of energy intake. The mean difference in 24-h MBG ranged from −0.10 to 1.19 mmol/L. Detailed characteristics of the interventions and outcomes, including 24-h MBG reported in both mmol/L and mg/dL, were summarized in Table 2, and prescribed energy intake for the intervention and control groups was provided in Supplementary Table 1.

**Table 2:** Article information extraction table.

Source of literature	Patients type	Total sample size	Age	Study duration	Number of meals provided by investigators (times/day)	Carbohydrate restriction level		24-hour MBG	
						Intervention group	Control group	Intervention group	Control group
Skytte et al. (2021) (13)	T2DM outpatients	28	64 ± 7.7	6 weeks	5 times/day^c^	CRHP: energy-percentage carbohydrate/protein/fat: 30%/30%/40%	Conventional diabetes diet	8.01 ± 0.22 (mmol/L)	9.2 ± 0.36 (mmol/L)
Al-Ozairi et al. (2023) (14)	T2DM outpatients	24	54 (47–56)	6 days	Number of meals provided according to participants’ habitual diet	Carbohydrate: 10%	Conventional diet^a^	7.4 ± 1.1(mmol/L)	7.3 ± 1.2 (mmol/L)
Chang et al. (2019) (21)	T2DM outpatients	46	59 ± 11	1 days	3 times/day	LCBF:<10% of energy from carbohydrate, 85% of energy from fat, 15% of energy from protein	Conventional diabetes diet	7.2 ± 1.1(mmol/L)	7.5 ± 1.5 (mmol/L)
Oliveira et al. (2023) (22)	T2DM outpatients	121	64 ± 9	12 weeks	1 time/day^d^	LC:~465 kcal: 25 g protein, 8 g carbohydrates, and 37 g fat	Conventional diabetes diet	7.0 ± 1.3(mmol/L)	7.6 ± 1.9 (mmol/L)
Thomsen et al. (2020) (23)	T2DM outpatients	32	64.0 (58.8–68.0)	2 days	5 times/day^c^	CRHP: carbohydrate/protein/fat:31%/29%/40%	Conventional diabetes diet	7.7 ± 1.6(mmol/L)	8.6 ± 2.0 (mmol/L)
Tay et al. (2014) (20)	T2DM outpatients	40	58 ± 7	24 weeks	Number of meals provided according to participants’ habitual diet	LC:14% carbohydrate [<50 g/day], 28% protein, and 58% fat [<10% saturated fat]	Conventional diabetes diet^b^	6.9 ± 1.2(mmol/L)	7.6 ± 1.8 (mmol/L)
Enyama et al. (2021) (24)	T2DM inpatients	38	61.4 ± 16.6	2 days	3 times/day	LCD for breakfast: 10% from carbohydrates, 25% from protein, and 65% from fat	Conventional diabetes diet	148 ± 28(mg/dL)^e^	166 ± 38 (mg/dL)

T2DM, type 2 diabetes mellitus; MD, mean difference; MBG, mean blood glucose.

a The comparator diet in Al-Ozairi et al. (14) was a habitual diet, not a standardized CD diet. No specific macronutrient composition was reported.

b Tay et al. divided participants by baseline mean glucose (>8.6 and ≤8.6 mmol/L). This meta-analysis only used data from the >8.6 mmol/L group.

c Five meals per day, including three main meals and pre- and post-dinner snacks.

d Only breakfast was provided by the study team; participants prepared all other meals themselves.

e values in mg/dL can be obtained by multiplying mmol/L by 18.

### 3.3 Assessment of study quality

**Figure 2:**
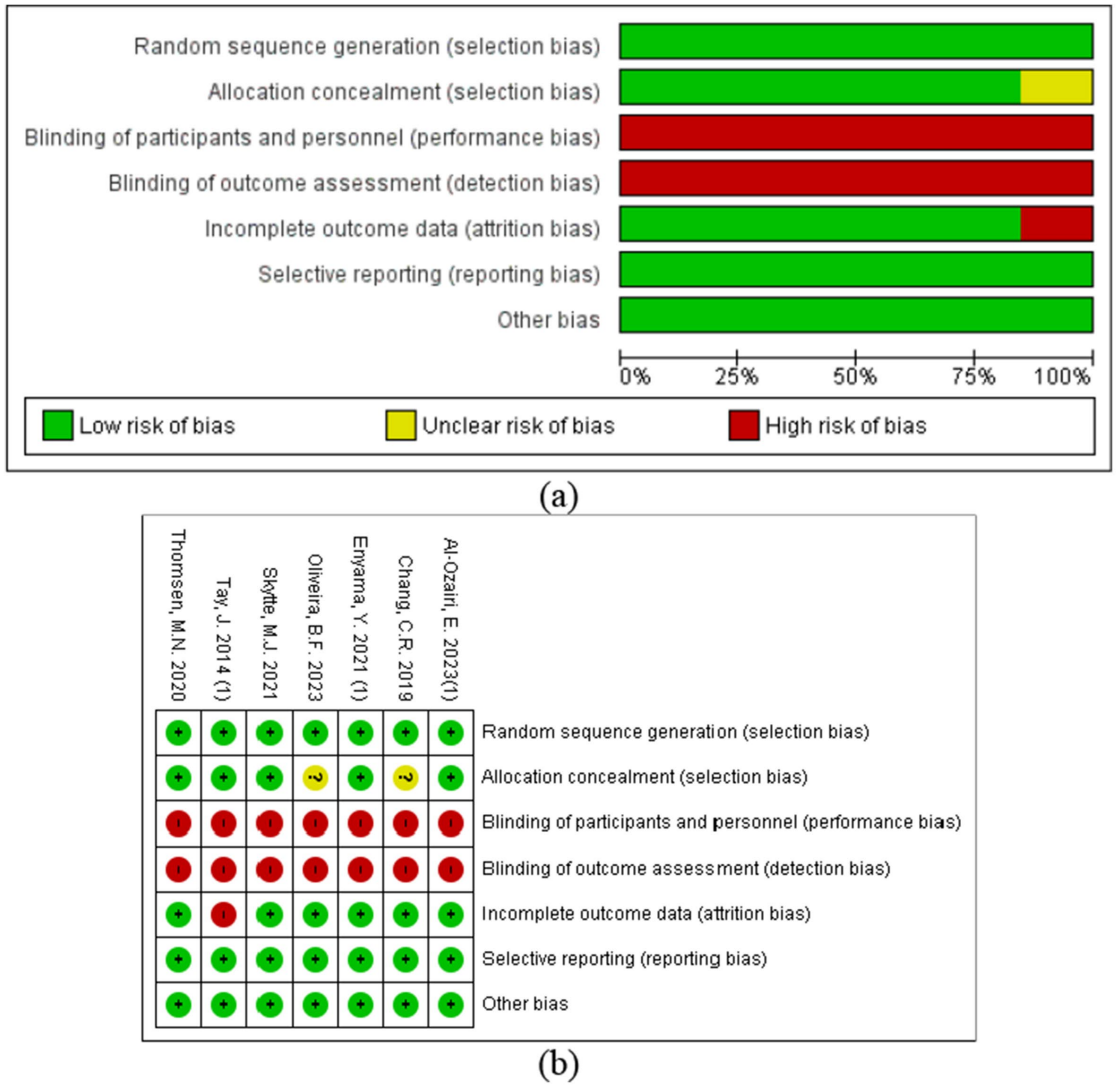
Risk of bias assessment of the included studies: **(a)** risk of bias graph; **(b)** risk of bias summary plot.

The quality of the included studies was evaluated, and all studies met the random sequence generation; 7 studies met the criteria for randomization (13, 14, 20–24); and 5 articles met the allocation concealment requirement (13, 14, 20, 23, 24). None of the included articles met the criterion for blinding of participants and personnel and blinding of outcome assessment. Furthermore, 6 articles met the criterion for incomplete outcome data (13, 14, 21–24); 7 articles met the criterion for selective reporting (13, 14, 20–24); and 7 articles met the criterion for other sources of bias (13, 14, 20–24) (see Figure 2 and Supplementary Table 3).

### 3.4 Effect size evaluation

**Figure 3:**
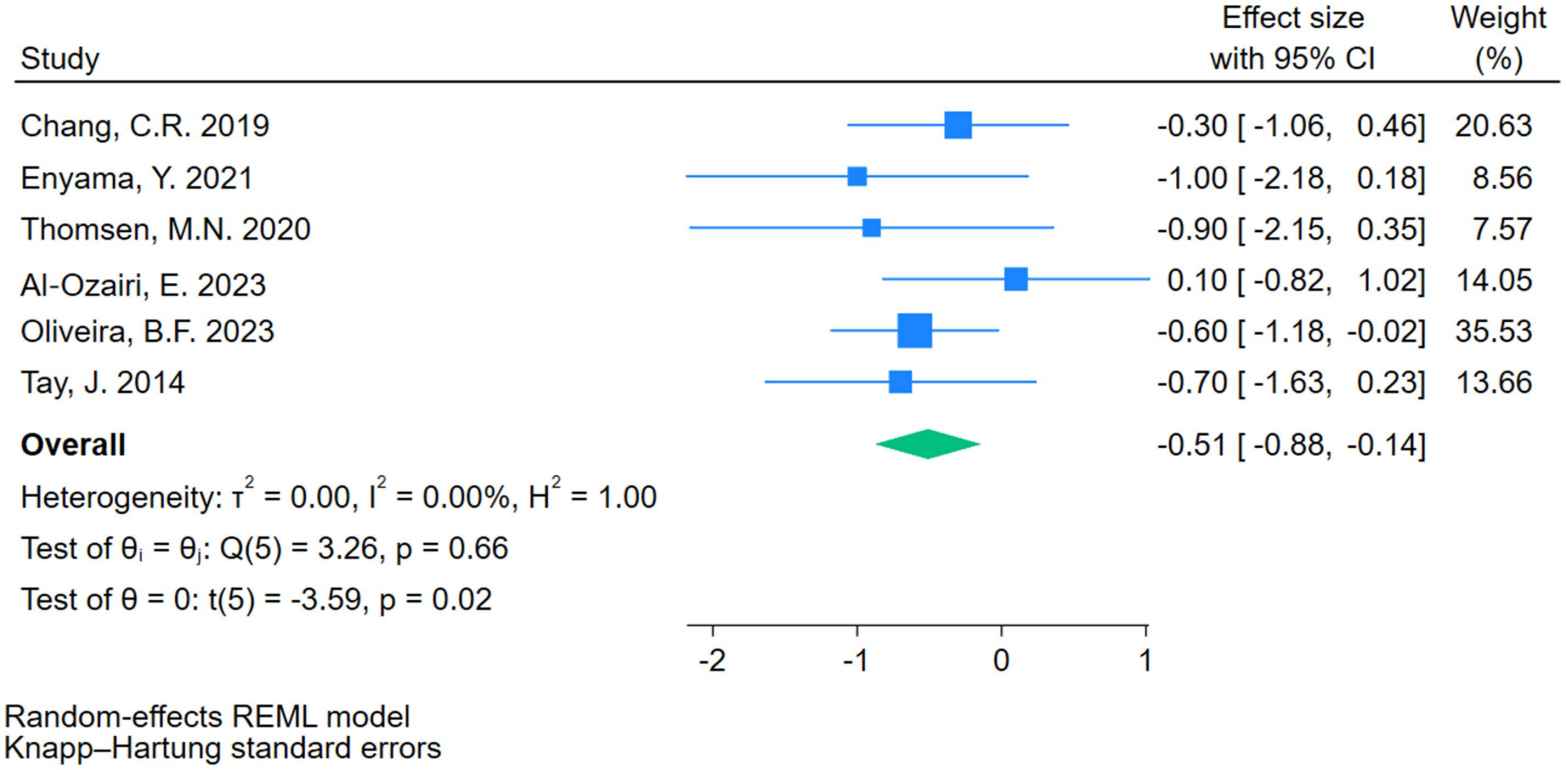
Forest plots showing the meta-analysis results after excluding Skytte et al. (13).

A total of seven RCTs involving 329 participants reported outcomes on 24-h MBG levels, with 162 patients in the intervention group and 167 in the control group (see Supplementary Figure 1). One study (13) reported incomplete CGM recordings, which contributed substantially to heterogeneity and may have biased the estimated 24-h MBG. After removing this study, heterogeneity decreased to I^2^ = 0% (*p* = 0.66) (see Figure 3). Given the small number of remaining studies, a random-effects model using REML was applied, and 95% confidence intervals were adjusted with the Hartung-Knapp method. The results indicated that CRDs significantly improved 24-h MBG levels in patients with T2DM (d = −0.51, 95% CI: −0.88 to −0.14, *p* < 0.05).

Due to the limited number of eligible comparisons, subgroup analyses and meta-regression were not conducted. The small sample size and wide confidence intervals suggest caution in interpretation.

### 3.5 Test for publication bias

**Figure 4:**
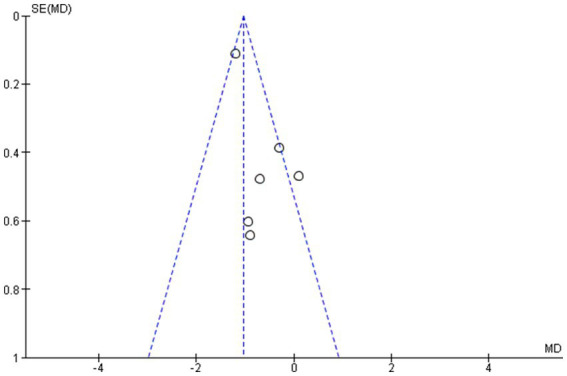
Publication bias funnel plot.

The outcome indicators of the included studies and the symmetrical distribution of scatter points on both sides of the funnel plot suggest that there is no publication bias. The publication bias analysis was performed based on the final set of studies after excluding one study with substantial heterogeneity (13). This exclusion was made to ensure a more accurate evaluation by minimizing the influence of potential outliers on the assessment of publication bias (see Figure 4).

### 3.6 Exploratory trend analysis

**Figure 5:**
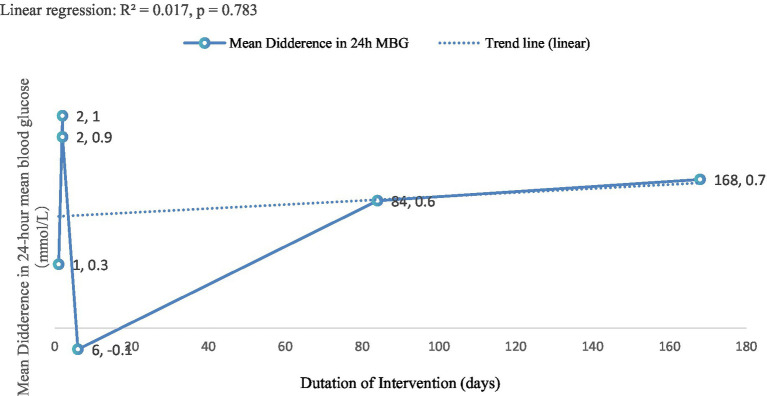
Trend analysis of intervention duration and 24-h MBG change. This exploratory trend analysis is limited by the small number of studies and should be interpreted cautiously.

Figure 5 illustrated the preliminary trend in 24-h mean blood glucose (24-h MBG) in patients with T2DM following carbohydrate-restricted dietary interventions of varying durations, which ranged from 1 to 168 days (24 weeks) (R^2^ = 0.017, *p* = 0.783). Most intervention durations were associated with reductions in 24-h MBG. For example, a 2-day intervention resulted in a 1.0 mmol/L decrease, while a 24-week intervention achieved a 0.70 mmol/L reduction. The only exception was observed at 6 days, which showed a slight increase of 0.10 mmol/L.

These findings suggested a potential association between longer intervention durations and greater improvements in glycemic control. The exploratory trend analysis further supported this observation, indicating a possible positive correlation between intervention duration and the magnitude of MBG reduction. Prolonged carbohydrate-restricted dietary interventions appeared to lead to more substantial improvements in 24-h MBG.

### 3.7 Sensitivity analysis

Sensitivity analysis was conducted by excluding studies one at a time to assess the robustness of the pooled results and to explore potential sources of heterogeneity. One study (13) was identified as substantially increasing heterogeneity (I^2^ from 0 to 52.07%) and was therefore excluded from the primary meta-analysis.

To specifically address the impact of study design, additional sensitivity analyses were performed by sequentially excluding the three crossover studies included in the meta-analysis: Al-Ozairi et al. (14), Chang et al. (21), and Thomsen et al. (23) (see Supplementary Figures 2–4). Excluding each of these studies individually did not materially alter the overall effect size or heterogeneity (I^2^ = 0%). Besides, exclusion of Oliveira et al. (22), in which only breakfast was provided by investigators while the remaining meals were self-prepared by participants, similarly had no impact on the pooled results. These findings indicate that the overall results are consistent, and all studies were retained in the final analysis.

In addition, sensitivity analyses related to the exploratory trend revealed that excluding either the shortest or the longest intervention duration separately did not affect the observed upward trend in the change of 24-h MBG, indicating robustness to single extreme values. However, when both the shortest and longest durations were excluded simultaneously, the trend was altered, suggesting that the observed association may be partially driven by the combined effect of these boundary data points.

## 4 Discussion

This study found that CRDs may improve 24-h MBG in patients with T2DM. Moreover, exploratory analysis suggested a potential positive association between the duration of carbohydrate-restricted dietary interventions and the magnitude of MBG improvement, indicating that longer intervention durations may confer greater glycemic benefits. The observed reduction of −0.51 mmol/L in 24-h MBG corresponds to an estimated HbA1c decrease of ~0.32%, which is clinically meaningful, as a 0.3% (3 mmol/mol) change in HbA1c is generally considered significant (25).

These effects may be partly related to changes in fat metabolism induced by CRDs. Due to insufficient energy supply from the CRDs, the body increasingly relies on lipolysis to produce energy, resulting in decreased blood glucose and insulin levels and the increase in plasma free fatty acids released from triglycerides (26). For example, Goday et al. (27) found that 4-month low carbohydrate diets significantly reduced weight by 14.7 kg, waist circumference by 12 cm, HbA1c by 0.9%, and Homeostasis Model Assessment for Insulin Resistance (HOMA index) by 3.4 in patients with T2DM, improving IS and glucose control. Besides, CRDs reduced dietary glucose intake, thereby attenuating overall glycemic fluctuations. Under these conditions, skeletal muscle requires less insulin, avoiding the development of insulin resistance associated with chronic hyperinsulinemia. This may help restore insulin sensitivity in skeletal muscle (28, 29). Luong et al. (30) found that 3 weeks ketogenic diet decreased weight by 2.2 kg, and increased glucose disposal during a hyperinsulinemic-euglycemic clamp, suggesting improved IS in skeletal muscle.

Sensitivity analyses excluding some studies did not materially change overall heterogeneity, but minor biases in MBG measurement remain possible. Although some studies (13, 14, 22) did not significantly increase statistical heterogeneity (I^2^), they may contribute to the over- or underestimation of 24-h MBG. For instance, Al-Ozairi et al. (14) controlled habitual carbohydrate intake at 10% but did not match total daily energy intake between intervention and control groups, which may limit the real-world representativeness of CGM measurements. Similarly, Oliveira et al. (22) controlled only breakfast carbohydrate content, leaving other meals uncontrolled, potentially biasing CGM outcomes. Skytte et al. (13) did not fully match carbohydrate intake across groups (intervention 2,502 KJ vs. control 2,504 KJ), which may slightly influence MBG measurements. Moreover, variations in macronutrient composition may affect LDL-C levels, which could confound the interpretation of intervention effects on glycemic outcomes (31). Thus, differences in total daily energy intake or macronutrient distribution may act as confounders, influencing CGM or glycemic outcomes. Also, many included studies had small sample sizes, which may limit the ability to detect potential biases and affect the robustness of the estimated effects.

However, it is important to note that the majority of data included in this meta-analysis were derived from very short-term interventions, typically lasting between 1 and 6 days. While these studies provide valuable insight into the acute effects of carbohydrate restriction on glycemic control, their relevance to the long-term management of T2DM was limited. Some studies indicated that long-term CRDs were ineffective. For example, Silverii et al. (32) reported that LCDs significantly reduced HbA1c at 3 and 6 months compared to high-carbohydrate diets. These benefits diminished over the medium to long term and reversed at 24 months, suggesting potential adverse effects potential adverse implications of long-term CRDs in individuals with T2DM. This conclusion was drawn from a meta-analysis of 37 randomized controlled trials involving 3,301 patients with type 2 diabetes (32). Similarity, Goldenberg et al. (31) reported that, compared to control diets, LCDs significantly increased rates of diabetes remission, reduced HbA1c levels, promoted weight loss, decreased triglycerides, and improved insulin sensitivity at 6 months. However, these benefits diminished by 12 months, and a worsening in quality of life and an increase in low-density lipoprotein cholesterol (LDL-C) levels were observed (31).

Given that T2DM was a chronic disease, long-term efficacy held limited clinical value (33, 34). As a result, major clinical guidelines—such as those from the American Diabetes Association (ADA) and the European Association for the Study of Diabetes (EASD)—did not recommend carbohydrate restriction diets for patients with T2DM (33, 35). The long-term implementation of CRDs in individuals with T2DM was limited by multiple factors, including dietary adherence and potential safety concerns (36, 37). Adherence frequently declines beyond 6–12 months due to the restrictive nature of the diet and reduced acceptability, which may attenuate or negate initial improvements in glycemic control and body weight (38). Additionally, elevations in LDL-C and uncertain long-term cardiovascular outcomes raised safety considerations, particularly among high-risk populations such as those with renal impairment, or concurrent use of SGLT2 inhibitors (1, 39). Accordingly, major guidelines, including ADA and EASD, did not recommend carbohydrate restriction as a standard long-term strategy, though short-term use may be considered under professional supervision (33, 35).

Although most meta-analyses have examined the effects of LCDs on T2DM patients across different intervention periods, the majority of included studies focus on short-term interventions, while long-term studies (1 year) remain limited. In addition, evidence directly comparing CGM outcomes between short- and long-term interventions was scarce. Therefore, it is necessary for future studies to systematically evaluate and compare the differential impacts of short- versus longer-term (1 year) CRDs on glycemic control.

### 4.1 Limitations

First, although some statistically significant differences were observed in glycemic outcomes between carbohydrate-restricted and balanced diets, the magnitude of these differences may lack clinical relevance. This limitation suggested that the practical impact of the intervention on patient health and long-term diabetes management requires cautious interpretation.

Second, the majority of studies included in this meta-analysis and related literature were conducted over very short durations, typically ranging from 2 to 6 days. Such short intervention periods may not adequately capture the effects of carbohydrate restricted diets on a chronic condition like T2DM, limiting the generalizability of these findings to long-term clinical practice.

Third, key clinical indicators such as time in range were not analyzed due to insufficient data across studies. Future high-quality RCTs with larger samples, longer durations, and standardized CGM reporting are warranted.

Fourth, although Figure 5 indicated a preliminary trend suggesting that longer carbohydrate-restricted interventions may modestly reduce 24-h MBG, the weak association (R^2^ = 0.017, *p* = 0.783) precludes firm conclusions. Future large-scale, long-term RCTs with standardized CGM outcomes are required to determine the clinical relevance of these observations.

Finally, although several trials reported the use of dietary logs or food records to monitor compliance, the original records were not accessible, which may limit the assessment of adherence.

## 5 Conclusion

CRDs may improve 24-h MBG in patients with T2DM, with exploratory trend analysis suggesting greater benefits with longer intervention durations. However, due to the limited number and relatively short duration of included studies, further high-quality randomized controlled trials with longer durations (≥1 year) are warranted to evaluate the differential effects of short-term and long-term CRDs on glycemic outcomes in patients with T2DM.

## Data Availability

The original contributions presented in the study are included in the article/Supplementary material, further inquiries can be directed to the corresponding author.
