# Supplementary material for: The effects of carbohydrate-restricted diets on 24-h mean blood glucose levels measured by continuous glucose monitoring in type 2 diabetes: a hypothesis-generating meta-analysis
Source: Front Nutr. 2025 Oct 7;12:1670022. doi: 10.3389/fnut.2025.1670022 (PMC12537420; doi:10.3389/fnut.2025.1670022)
Supplement: Supplementary file 1 [file Table_1.docx]

Supplementary Material

# Figures and Tables

## Figures


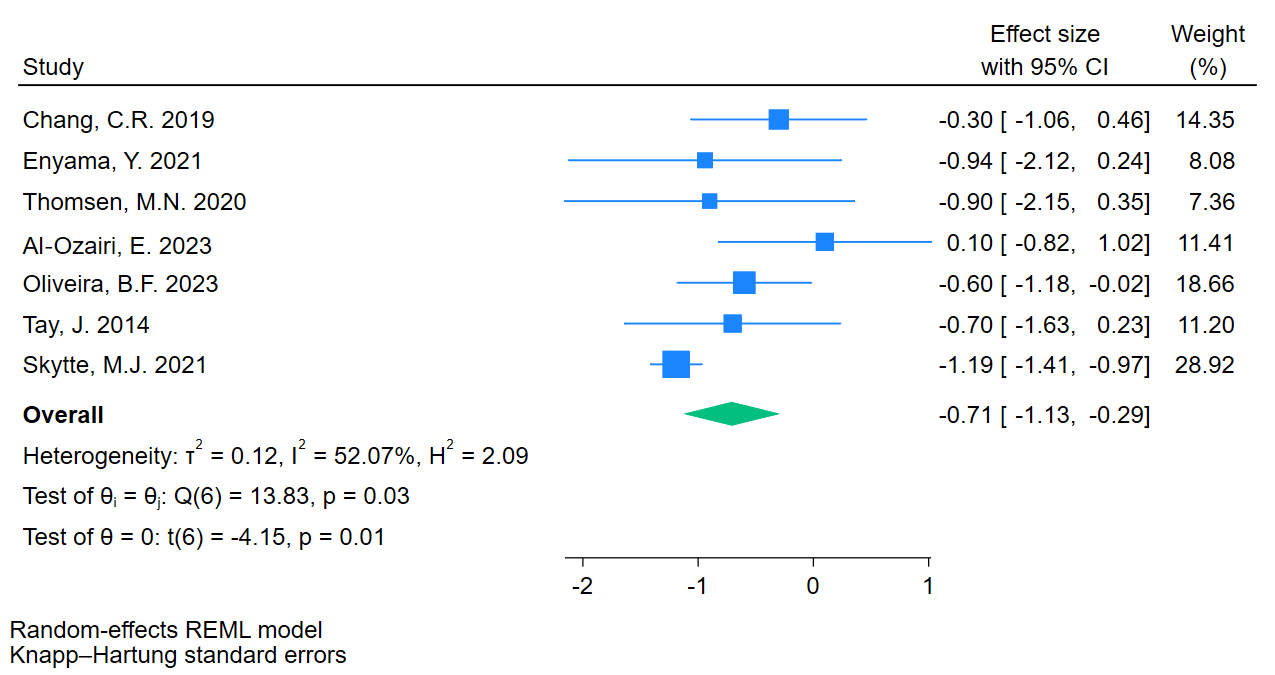


**FIGURE 1.** Forest plots showing the effect of carbohydrate-restricted diets on 24-hour MBG in patients with T2DM.


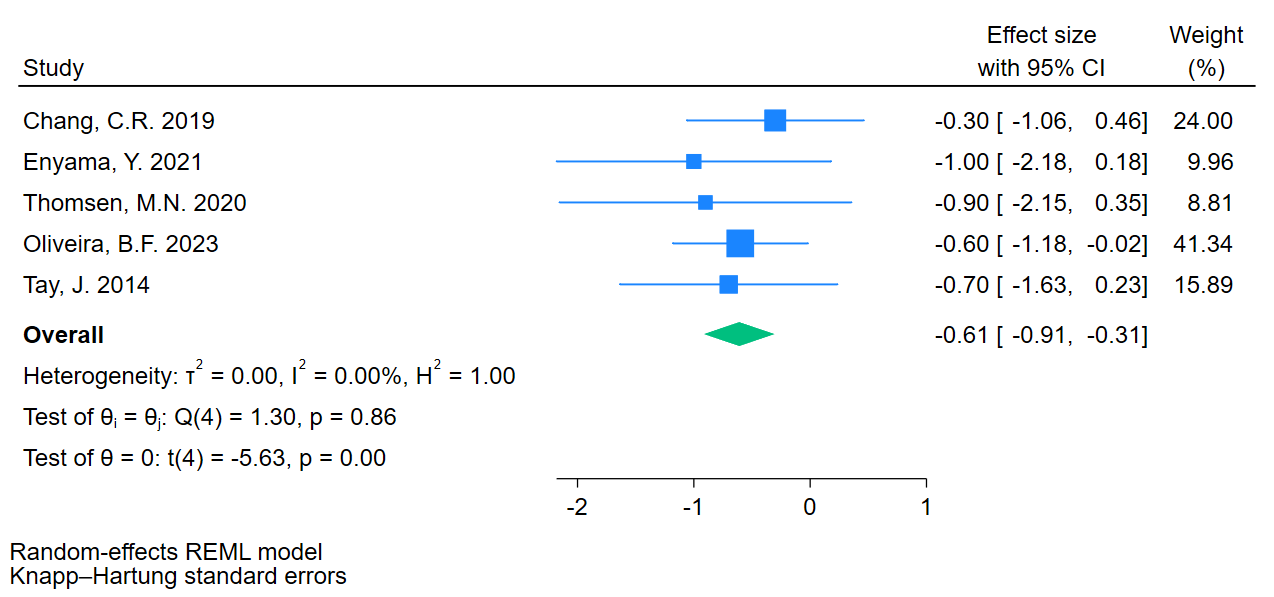


**FIGURE 2.** Forest plots showing the meta-analysis results after excluding Al‐Ozairi, E. 2023.


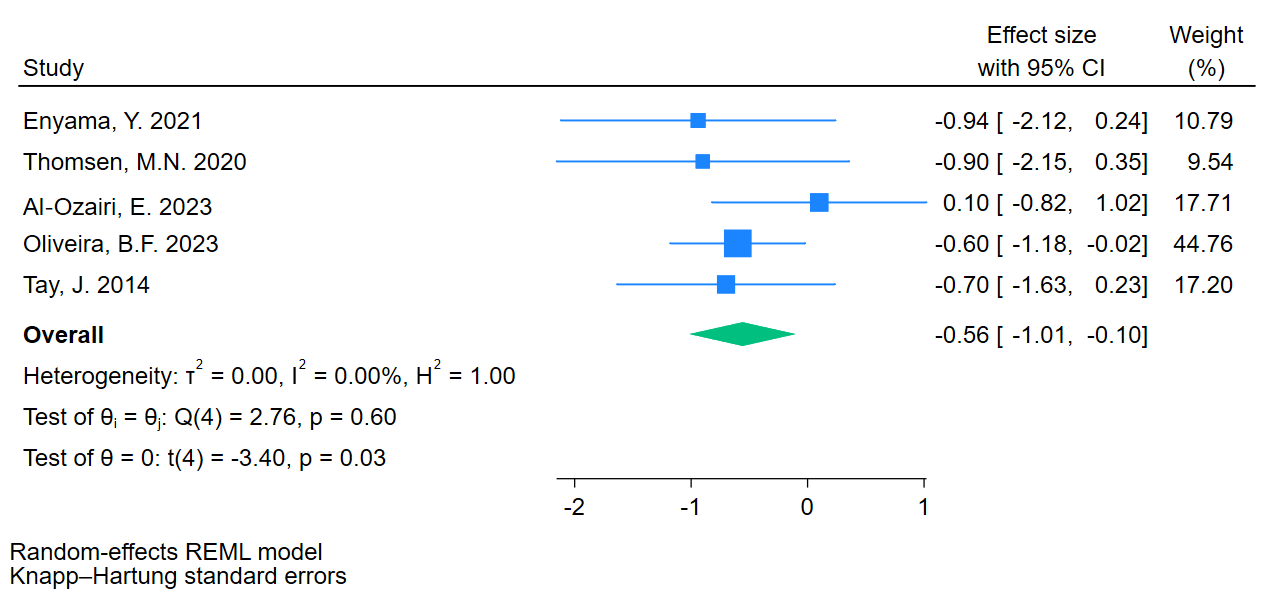


**FIGURE 3.** Forest plots showing the meta-analysis results after excluding Chang, C.R. 2019.

**
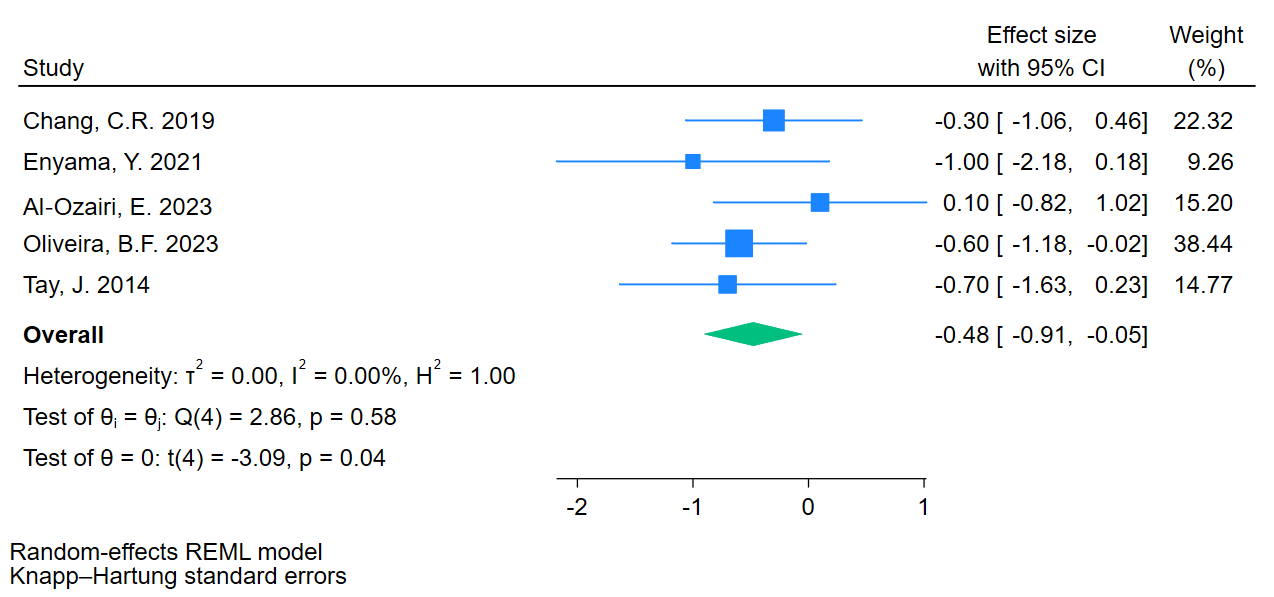
**

**FIGURE 4.** Forest plots showing the meta-analysis results after excluding Thomsen, M.N. 2020.

## Tables

**Table 1**. Prescribed total daily energy intake in intervention and control groups across included studies.

| Source of literature | Energy matching type | Energy content | MD in 24-hour MBG(mmol/L) |
| --- | --- | --- | --- |
| Chang, C.R. 2019 | Total daily energy matched | 1921 ± 387 kcal | 0.3 |
| Oliveira, B.F. 2023 | Breakfast only | 450 kcal (breakfast only) | 0.6 |
| Skytte, M.J. 2021 | Per meal & total daily energy matched | CRHP: 2502 KJ; CD: 2504 KJ | 1.19 |
| Thomsen, M.N. 2020 | Total daily energy matched | 10298 kJ) | 0.9 |
| Al-Ozairi, E. 2023 | Not reported | Habitual diet | -0.1 |
| Tay, J. 2014 | Total daily energy matched | 1429 kcal | 0.7 |
| Enyama, Y. 2021 | Total daily energy matched | 30 kcal/kg/day | 1 |

Abbreviation: T2DM, type 2 diabetes mellitus; MD, mean difference; MBG, mean blood glucose.

**Table 2.** AMSTAR-2 Assessment of Methodological Quality of Included Studies.

| **AMSTAR 2**  **1. Did the research questions and inclusion criteria for the review include the components of PICO?**   \| □ Population  For Yes:  Optional (recommended)  □ Timeframe for follow-up  ☑  □  Yes  No  □ Intervention  □ Comparator group  □ Outcome \| \| --- \|   **2. Did the report of the review contain an explicit statement that the review methods were**  **established prior to the conduct of the review and did the report justify any significant deviations from the protocol?**   \| For Yes:  As for partial yes, plus the protocol should be registered and should also have specified:  □ a meta-analysis/synthesis plan, if appropriate, *and*  □ a plan for investigating causes of heterogeneity  □ justification for any  deviations from the protocol  ☑ Yes  □ Partial Yes □ No  For Partial Yes:  The authors state that they had a written protocol or guide that included ALL the following:  □ review question(s)  □ a search strategy  □ inclusion/exclusion criteria  □ a risk of bias assessment \| \| --- \|   **3. Did the review authors explain their selection of the study designs for inclusion in the review?**   \| For Yes, the review should satisfy ONE of the following:  □  ☑  Yes  No  □ *Explanation for* including only RCTs  □ OR *Explanation for* including only NRSI  □ OR *Explanation for* including both RCTs and NRSI \| \| --- \|   **4. Did the review authors use a comprehensive literature search strategy?**   \| For Yes, should also have (all the following):  For Partial Yes (all the following):  □ searched at least 2 databases (relevant to research question)  □ provided key word and/or search strategy  □ justified publication  restrictions (eg, language)  □  ☑  □  Yes  Partial Yes No  □ searched the reference lists/bibliographies of included studies  □ searched trial/study registries  □ included/consulted content experts in the field  □ where relevant, searched for grey literature  □ conducted search within 24 months of completion of the review \| \| --- \|   **5. Did the review authors perform study selection in duplicate?**   \| For Yes, either ONE of the following:  ☑  □  Yes  No  □ at least two reviewers independently agreed on selection of eligible studies and achieved consensus on which studies to include  □ OR two reviewers selected a sample of eligible studies and achieved good agreement (at least 80 per cent), with the remainder selected by one reviewer \| \| --- \|   **6. Did the review authors perform data extraction in duplicate?**   \| For Yes, either ONE of the following:  □ at least two reviewers achieved consensus on which data to extract ☑ Yes \| \| --- \| |
| --- | --- | --- | --- | --- | --- | --- |

| \| from included studies  □ No  □ OR two reviewers extracted data from a sample of eligible studies and achieved good agreement (at least 80 per cent), with the remainder  extracted by one reviewer \| \| --- \|   **7. Did the review authors provide a list of excluded studies and justify the exclusions?**   \| For Partial Yes:  □ provided a list of all  potentially relevant studies  that were read in full text form but excluded from the review  □ Yes  □ Partial Yes ☑ No  For Yes, must also have:  □ Justified the exclusion from the review of each  potentially relevant study \| \| --- \|   **8. Did the review authors describe the included studies in adequate detail?**   \| For Yes, should also have ALL the following:  □ described population in detail  □ described intervention and comparator in detail  (including doses where relevant)  □ described study’s setting  □ timeframe for follow-up  □ Yes  ☑ Partial Yes □ No  For Partial Yes (ALL the following):   \| □  □  □  □  □ \| described described  described described  described \| populations  interventions  comparators  outcomes  research designs \| \| --- \| --- \| --- \| \| \| --- \| --- \| --- \| --- \|   **9. Did the review authors use a satisfactory technique for assessing the risk of bias (RoB) in individual studies that were included in the review?**   \| **RCTs**  For Partial Yes, must have assessed RoB from  For Yes, must also have assessed RoB from:  □ allocation sequence that was ☑ Yes  not truly random, *and* □ Partial Yes  □ selection of the reported □ No  result from among multiple □ Includes only  measurements or analyses of NRSI  a specified outcome  □ unconcealed allocation, *and*  □ lack of blinding of patients  and assessors when assessing outcomes (unnecessary for  objective outcomes such as all cause mortality) \| \| --- \| \| **NRSI**  For Partial Yes, must have assessed RoB:  For Yes, must also have assessed RoB:  □ methods used to ascertain exposures and outcomes, *and*  □ selection of the reported  result from among multiple measurements or analyses of a specified outcome  □ Yes  □ Partial Yes □ No  □ Includes only RCTs  □ from confounding, *and*  □ from selection bias \|   **10. Did the review authors report on the sources of funding for the studies included in the review?**   \| For Yes  □ Must have reported on the sources of funding for individual studies included in the review. Note: Reporting that the reviewers looked for this information but it was not reported by study authors also qualifies  □  ☑  Yes  No \| \| --- \|   **11. If meta-analysis was performed did the review authors use appropriate methods for statistical combination of results?**   \| **RCTs**  ☑ Yes □ No  □ No meta-analysis  For Yes:  □ The authors justified combining the data in a meta-analysis  □ AND they used an appropriate weighted technique to combine study results and adjusted for heterogeneity if present \| \| --- \| |
| --- | --- | --- | --- | --- | --- | --- | --- | --- | --- | --- |

| \| □ AND investigated the causes of any heterogeneity conducted \| \| --- \| \| **For NRSI**  □ Yes □ No  □ No meta-analysis conducted  For Yes:  □ The authors justified combining the data in a meta-analysis  □ AND they used an appropriate weighted technique to combine study results, adjusting for heterogeneity if present  □ AND they statistically combined effect estimates from NRSI that were adjusted for confounding, rather than combining  raw data, or justified combining raw data when adjusted effect estimates were not available  □ AND they reported separate summary estimates for RCTs and NRSI separately when both were included in the review \|   **12. If meta-analysis was performed, did the review authors assess the potential impact of RoB in individual studies on the results of the meta-analysis or other evidence synthesis?**   \| For Yes:  ☑ Yes □ No  □ No meta-analysis conducted  □ included only low risk of bias RCTs  □ OR, if the pooled estimate was based on RCTs and/or NRSI at variable RoB, the authors performed analyses to investigate possible impact of RoB on summary estimates of effect \| \| --- \|   **13. Did the review authors account for RoB in individual studies when interpreting/discussing the results of the review?**   \| For Yes:  ☑  □  Yes  No  □ included only low risk of bias RCTs  □ OR, if RCTs with moderate or high RoB, or NRSI were included the  review provided a discussion of the likely impact of RoB on the results \| \| --- \|   **14. Did the review authors provide a satisfactory explanation for, and discussion of, any heterogeneity observed in the results of the review?**   \| For Yes:  ☑  □  Yes  No  □ There was no significant heterogeneity in the results  □ OR if heterogeneity was present the authors performed an investigation of sources of any heterogeneity in the results and discussed the impact of this on the results of the review \| \| --- \|   **15. If they performed quantitative synthesis did the review authors carry out an adequate**  **investigation of publication bias (small study bias) and discuss its likely impact on the results of the review?**   \| For Yes:  ☑  □  □  □ performed graphical or statistical tests for publication bias and  discussed the likelihood and magnitude of impact of publication bias  Yes  No  No meta-analysis conducted \| \| --- \|   **16. Did the review authors report any potential sources of conflict of interest, including any funding they received for conducting the review?**   \| For Yes:  ☑  □  Yes  No  □ The authors reported no competing interests OR  □ The authors described their funding sources and how they managed potential conflicts of interest \| \| --- \| |
| --- | --- | --- | --- | --- | --- | --- | --- |

**Table 3.** Tables for risk of bias.

| Study (Author, Year) | Random sequence generation | Allocation concealment | Blinding of participants and personnel | Blinding of outcome assessment | Incomplete outcome data | Selective reporting | Other bias |
| --- | --- | --- | --- | --- | --- | --- | --- |
| Chang, C.R. 2019 | Low risk | Middle risk | High risk | High risk | Low risk | Low risk | Low risk |
| Oliveira, B.F. 2023 | Low risk | Middle risk | High risk | High risk | Low risk | Low risk | Low risk |
| Skytte, M.J. 2021 | Low risk | Low risk | High risk | High risk | Low risk | Low risk | Low risk |
| Thomsen, M.N. 2020 | Low risk | Low risk | High risk | High risk | Low risk | Low risk | Low risk |
| Al-Ozairi, E. 2023 | Low risk | Low risk | High risk | High risk | Low risk | Low risk | Low risk |
| Tay, J. 2014 | Low risk | Low risk | High risk | High risk | High risk | Low risk | Low risk |
| Enyama, Y. 2021 | Low risk | Low risk | High risk | High risk | Low risk | Low risk | Low risk |
